# Evolution of Analysis of Polyhenols from Grapes, Wines, and Extracts

**DOI:** 10.3390/molecules18011076

**Published:** 2013-01-16

**Authors:** Bénédicte Lorrain, Isabelle Ky, Laurent Pechamat, Pierre-Louis Teissedre

**Affiliations:** 1 Université Bordeaux, ISVV, EA 4577Oenologie, F-33140 Villenave d’Ornon, France; 2 INRA, ISVV, USC1366Oenologie, F-33140 Villenave d’Ornon, France

**Keywords:** phenolics, anthocyanins, tannins, extraction, global analyses, HPLC/UPLC, spectroscopy, DPm

## Abstract

Grape and wine phenolics are structurally diverse, from simple molecules to oligomers and polymers usually designated as tannins. They have an important impact on the organoleptic properties of wines, that’s why their analysis and quantification are of primordial importance. The extraction of phenolics from grapes and from wines is the first step involved in the analysis. Then, several analytical methods have been developed for the determination of total content of phenolic, while chromatographic and spectrophotometric analyses are continuously improved in order to achieve adequate separation of phenolic molecules, their subsequent identification and quantification. This review provides a summary of evolution of analysis of polyphenols from grapes, wines and extracts.

## 1. Introduction

Phenolic compounds are the most abundant secondary metabolites present in the plant kingdom. They possess a common structure comprising an aromatic benzene ring with one or more hydroxyl substituents. They represent a large and diverse group of molecules including two main families: the flavonoids based on common C6-C3-C6 skeleton and the non-flavonoids. In plant, they play a role in growth, fertility and reproduction and in various defence reactions to protect against abiotic stress like UV-light or biotic stresses such as predator and pathogen attacks [1,2]. They also constitute basic components of pigments, essences and flavors.

Many of phenolic compounds (resveratrol, quercetin, rutin, catechin, proanthocyanidins) have been reported to have multiple biological activities, including cardioprotective, anti-inflammatory, anti-carcinogenic, antiviral and antibacterial properties attributed mainly to their antioxidant and antiradical activity [3,4,5,6].

Phenolic compounds are essential for the quality of plant-derived food products through their contribution to oxidative stability and organoleptic characteristics. Indeed, wine organoleptic properties are largely related to phenolic compounds extracted from the grape during the winemaking process. Among them, flavonoids, including anthocyanins and flavan-3-ols, are the most important for wine quality. Anthocyanins are pigmented compounds responsible for the red wine colour and they are essentially located in grape skins. Flavan-3-ols exist not only as monomers but also as oligomers and polymers, called condensed tannins or proanthocyanidins. Condensed tannins are grape-derived compounds of great importance to red wine quality due to their astringent, bitter properties [7,8] and their role in the long-term color stability [9,10]. Astringency and bitterness are two major characteristics in grape and wine quality definition. Astringency is a tactile sensation, whereas bitterness is a taste. The molecular size of proanthocyanidins affects their relative bitterness and astringency level [7,8,11,12]. Overall, monomers are more bitter than astringent, whereas the reverse is true in the case of large molecular weight derivatives. For grape seed tannin, reducing the degree of galloylation only decreases astringency [11].

The determination of the quantitative composition and the investigation of the factors affecting the composition of these bioactive substances, using robust, sensitive and reliable methods are considered a priority. Some common structures (catechin, proanthocyanidin, anthocyanins, *etc*.) have been already identified and quantified in wines but others ones such as high molecular mass phenolics or new formed compounds during wine ageing still remain to study. Many different methods have been improved through years. General approaches allow the determination of a global index (e.g., “total polyphenols”) mainly achieved by spectrophotometric detection and are opposed to more specific analyses based on separation of the individual polyphenolic species typically by high-performance liquid chromatography or capillary electrophoresis and their subsequent detection by different detectors, UV-vis, mass spectrometry.

This review is aimed to take stock on the different methods developed in the last years and the development of new ones. Examples of the new molecules they allowed to identify and quantify in grapes and wines will be given.

## 2. Structures of Main Polyphenols from Gapes and Wines

Wine phenolic composition depends on the grape used and on winemaking processes that determine their extraction into the must and subsequent reactions. Structures of phenolic compounds include simple aromatic ring with low molecular weight to complex high molecular weight tannins. Two groups of phenolic compounds are classically distinguished: flavonoids based on common C6-C3-C6 skeleton and non flavonoids.

**Figure 1:**
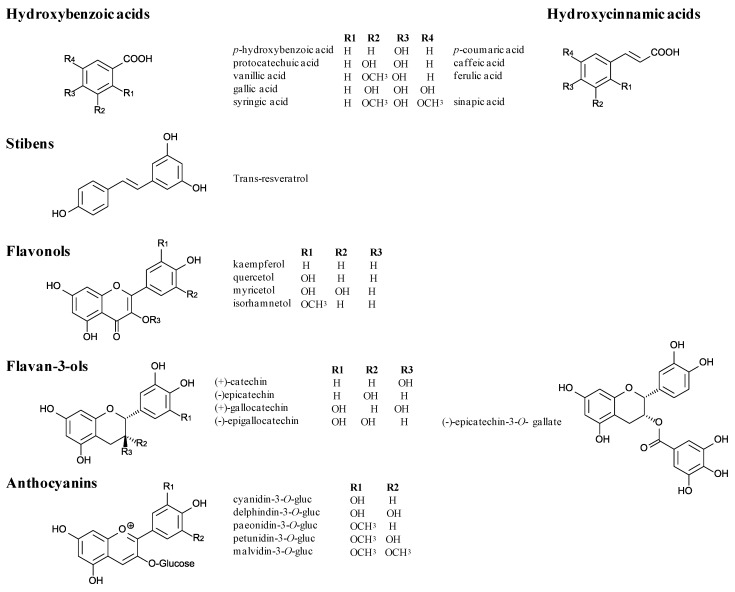
Structures of important monomeric phenolic compounds in grapes and wines.

Among non-flavonoids, principal compounds are phenolic acids (hydroxybenzoic acids), hydroxycinnamic acids and stilbens. Hydroxybenzoic acids are based on a C6-C1 structure, a benzene ring with one carbon aliphatic chain substituent. The various acids are differentiated by the substitution of their benzene ring. Vanillic, syringic and gallic acids are the main compounds from this sub-class (Figure 1). Several hydroxycinnamic acids (C6-C3) are present in grapes and wines (Figure 1). They have been identified in small quantities in the free form, but are mainly esterified, in particular with tartaric acid [13]. They may also be simple glycosides of glucose. Another family of more complex polyphenols is also present in grapes, wine and oak wood. Stilbenes have two benzene rings, generally bonded by an ethane, or possibly ethylene, chain. Among these *trans*-isomer compounds, resveratrol, or 3,5,4-trihydroxystilben (Figure 1), is thought to be produced by vines in response to a fungal infection [14].

Flavonoids, the most abundant phenolic compounds in grapes and wines, possess a common C15-skeleton, composed of three rings (A, B, C). This molecules family is constituted by different sub-categories, flavones, flavonols, flavanones, flavanols and anthocyanins differing by the ring C insaturation degree and substituents (Figure 1).

**Figure 2:**
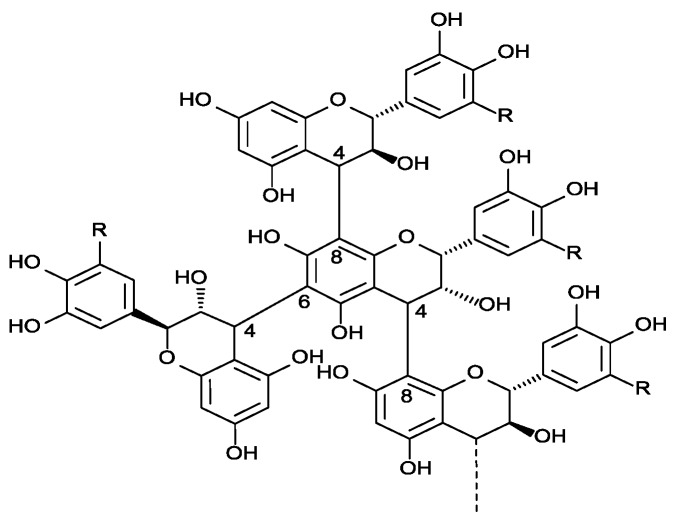
General structures of proanthocyanidins: flavan-3-ol monomers are linked through C4-C8 or C4-C6 linkages.

In this review, we will pay particular attention on anthocyanins and flavanols or proanthocyanidins. Anthocyanins are specific to red varieties and localized in berry skins except in teinturier varieties that have colored flesh. Their structure, flavylium cation, includes two benzene rings bonded by an unsaturated cationic oxygenated heterocycle, derived from the 2-phenyl-benzopyrylium nucleus (Figure 1). They are glucosylated derivatives of five aglycones or anthocyanidins: cyanidin, peonidin, petunidin, delphinidin and malvidin. Further diversity results from acylation of the glucose by acetic, p-coumaric and caffeic acids. Flavan-3-ols formed the more complex flavonoid sub-family (Figure 1). These compounds include simple monomers such as (+)-catechin and (−)-epicatechin but also oligomers or polymers called proanthocyanidins because they release anthocyanidins when heated in acidic solutions (Figure 2). Proanthocyanin structures vary in the nature of their constitutive sub-units, mean degree of polymerization (mDP) and linkage position.

They are located in all the parts of a grape cluster but skins contain lower amounts of proanthocyanidins than seeds and their structural characteristics also differ. Grape seed proanthocyanidins comprise only procyanidins [subunits constituted of (+)-catechin (C) and (−)-epicatechin (EC)], whereas grape skin proanthocyanidins include both procyanidins and prodelphinidins [subunits constituted of (−)-epigallocatechin (EGC)] [15]. Skin proanthocyanidins have a higher mDP and a lower proportion of galloylated subunits than seed ones. Condensed tannins are grape-derived compounds of great importance to red wine quality due to their astringent, bitter properties [7,8] and their role in the long-term color stability [9,10].

## 3. Extraction from Grapes

For characterizing phenolic compound structures, the anthocyanins or tannins need first to be extracted from grape. Regarding wine, some authors estimate that sample don’t need any particular preparation and can be directly injected or experimented while others indicate a great benefit on separation/quantification/identification after a purification step (this point will be considered in the analysis part).

### 3.1. Liquid-Solid Extraction

In several studies, grape seeds and skins are first separated before being separately extracted with solvents such as aqueous acetone and sometimes following by a second extraction with aqueous methanol [16,17,18]. Anthocyanins/anthocyanidins are usually extracted from skins or pomaces with acidified organic solvents, commonly methanol. More and more, these “manual” solid-liquid extractions are replaced by alternative extractions with pressurized liquid, ultrasound, electrical and microwaves-assisted extractions. These “mechanical” techniques are explored in recent scientific studies in order to shorten the extraction time, decrease the solvent consumption, increase the extraction yield and enhance the quality of extracts [19,20,21,22,23,24,25,26]. The pressurized liquid extraction (PLE) commercially known as accelerated solvent extraction (ASE) enables rapid extraction of analytes in a closed and inert environment under high pressure (3.3–20.3 MPa) and temperatures (40–200 °C). The major advantage of this technique is that pressurized solvent remain in liquid state well above their boiling points, allowing for high-temperature extraction improving analyte solubility and desorption kinetics from the matrices. Hence, extraction solvents including water that are inefficient in extracting phytochemicals at low temperatures may be much more efficient at elevated PLE temperatures. Indeed, Ju and Howard showed that high-temperature (80–100 °C) PLE using acidified water was as effective as acidified 60% methanol in extracting anthocyanins from grape skins [24]. In other works, high yields of total polyphenols and total flavonoids from Pinot noir grape skins were obtained working at 150 °C even though flavonoids have the tendency to degrade when extraction time is prolonged (more than 210 min) [22]. Pineiro *et al*. showed that PLE extraction of grape seeds using methanol as solvent produces results in terms of recovery of catechin and epicatechin, notably higher than those ones obtained by magnetic stirring or ultrasound-assisted extraction [23].

A second alternative extraction technique concerned electrically assisted extraction. Boussetta and co-workers showed that extraction of Chardonnay grape skins by pulsed electric field (PEF) and particularly high-voltage electrical discharge (HVED) treatments allowed acceleration of the extraction kinetics of the soluble matter and polyphenols in water at 20 °C [21]. HVED application introduces complex phenomena like shock waves and cavitation, which cause mechanical damage of grape tissues and disintegration of cell walls. Thus, an increase in polyphenol concentration reflecting the enhancement of diffusion between the cells at the inner surface of grape skins during the PEF or HVED treatment was also noticed by Boussetta and co-workers. However, these authors underlined a noticeable selectivity in polyphenol extraction and mentioned the necessity of further studies on this topic to improve this promising technique. Effectively, some parameters such as the number of discharges have to be optimized because too long treatments can impact negatively polyphenol extraction (decrease in concentration) possibly linked to their degradation [19]. Moreover, these two studies were performed on catechin or total phenolic content and questions concerning extraction of more sensitive oxidative molecules such as anthocyanins have been raised.

Finally microwave-assistance provided to be another very rapid and efficient mean of extraction [20]. This technique offers a rapid delivery of energy to a total volume of solvent and liquid with subsequent heating of the solvent and solid matrix, efficiently and homogeneously [27]. Application of optimal conditions to grape seed from Cabernet-Sauvignon, Shiraz, Sauvignon blanc and Chardonnay revealed that approximately 92% of the total polyphenols were extracted which was comparable or better than other extraction methods (ultrasounds, …) [26].

### 3.2. Extract Purification

These solid-liquid extractions lead to a “crude” extract which purity and specificity can still be improved. Thus, further fractionation by means of liquid/liquid extraction can be achieved to purify and to separate different procyanidins molecular weight fractions. Thus, after removing lipophilic materials by chloroform, Lorrain and co-workers employed ethyl acetate to obtain a monomeric/oligomeric proanthocyanidin fraction in the organic phase while polymers were concentrated in aqueous fraction [28]. More distinctive fractions (seven fractions from DPm 3 to 12) can be obtained by successive liquid/liquid extraction with increasing percentages of chloroform in methanol [29]. Alternatively, it is possible to enrich and fractionate crude extracts by semi-preparative normal phase LC (C18) [30] or by solid phase extraction employing diverse sorbents such as C18, XAD or PVPP [31,32,33]. These aspects will be further discussed, in the part “Techniques to establish tannin structures (mean degree of polymerization)”.

### 3.3. Phenolic Extraction from Grape to Wine: Methods for Prediction

Both the quantity and the extractability of anthocyanins and tannins increase throughout the grape ripening. A great number of different methods have been proposed to determine the phenolic ripening of grapes and to predict the wine phenolic composition of wines but up to now, none universal method was accepted [34,35,36,37,38]. The main variables that influence the yield and rate of phenolic extraction from grapes are particle size, temperature, maceration time, pH, solvent-to-solid ratio and type of solvent used. Indeed, Glories suggested a rapid, fairly simple method, giving results that are both comprehensive and easy to interpret [34]. The principle of this assay consists of rapidly extracting (4 h) the anthocyanins from the skins, in condition approximately comparable to that occurring in fermentation vats at pH 3.2 (solution with 5 g/L tartaric acid) and then under more extreme conditions, where all of the anthocyanins are then extractable and solubilized at pH 1. This method conducts in several parameters, ApH 1, the total potential in anthocyanins, ApH 3.2, the total potential in extractible anthocyanins, EA, the anthocyanin extractability, RPT, the total polyphenolic richness of the grapes and MP%, the seed maturity, representative of the contribution of seed tannins. More recently, FT-MIR spectroscopy combined with PLS statistical analyses was shown to be a fast and reliable technique for monitoring the phenolic ripening in grapes during the harvest period [37]. The same authors also presented a fast and simple extraction method of red grapes making possible to obtain good correlations between phenolic parameters of the grape extracts and those of their corresponding wines [35].

## 4. Analyses of Wines and Extracts

They are two general approaches to examine and to quantify the polyphenolic content in extracts and in wines. The determination of a total index by spectrophotometric detection or the separation of the individual phenol species and their subsequent detection.

### 4.1. Global Phenolic Determination Methods

A number of spectrophotometric methods for quantification of phenolic compounds in plant materials have been developed. These assays are based on different principles and are used to determine different structural groups present in phenolic compounds. They are well described in several reviews [31,39]. Briefly, the easiest method for a quickly estimation of the total phenolic compound in a wine or an extract is the measurement of absorption at 280 nm (with an appropriate sample dilution). This value is based on the characteristic absorption of the benzene cycles of the majority of phenols at 280 nm. This test presents a number of advantages, including speed and reproducibility. However, certain molecules, such as cinnamic acids and chalcones, have no maximum absorption at this wavelength. However, as they are present in wine at very low concentrations, any error in the value will be very small. Reversely other non phenolic molecules can possess a benzene ring (amino acid) and absorb at 280 nm conducting in interference absorption. A second method for a global phenolic content determination is the Folin-Ciocalteu assay which acts on the phenols due to their reductive properties. Indeed, it consists in the reduction of phosphomolybdic acid to a blue colored complex by phenolic compounds in alkaline conditions. However, this method remains not specific since some phenolic groups found in extractable proteins or reducing substances such as ascorbic acid can also participate in the reduction reaction. More specific assay such as vanillin assay, DMACH assay, Bate-Smith assay have been proposed for determining the content of proanthocyanidic tannins. Vanillin and DMACH assays rely on the formation of coloured products from the reaction between tannins and the aldehyde reagent while Bate-Smith principle is based on proanthocyanidin depolymerization through the breakdown of their intra-flavonols bonds in an acidic heat medium [40]. This results in carbocations formation partially converted into red cyanidins (with a specific absorption at 550 nm) if the medium is sufficiently conducive to oxidation [41]. Other methods are based on the selective precipitation of tannin by proteins (e.g., bovine serum albumin) or by other reagents such as polymers (polyethyleneglycol, polyvinylpyrrolidone, …). Methyl cellulose, a form of polysacharride, was also used to develop a method by precipitation for the quantification of condensed tannins in red wines or grape extracts, referred to as the MCP (methyl cellulose precipitation) tannin assay [42]. This assay is based on polymer-tannin interactions, resulting in an insoluble complex which precipitates. It is a substractive method since measurements are performed at 280 nm before and after precipitation. In general, all these assays are dependent on many variables including pH, isoelectric point, ionic strength, protein conformation and temperature. However, these methods lack of specifity and reproducibility because they are hindered by our inability to measure directly the removed tannins as the ideal absorption for spectral quantification at 280 nm suffers interference from the added protein precipitant [31]. Finally, regarding anthocyanins quantification, the chemical methods are based on the specific properties of anthocyanins: color variation according to pH and bleaching by sulfur dioxide [43].

Traditional spectroscopic assays may lead to overestimation of polyphenols contents of crude extracts because of the possible interference by UV-absorbing substances. Moreover, they can be time and solvent consuming (Folin, Bate-Smith). New approaches are being studied in order to develop novel, easy, reliable and robust global methods. Among them, electroanalytical techniques and infrared spectroscopy appeared to be the most promising.

**Figure 3:**
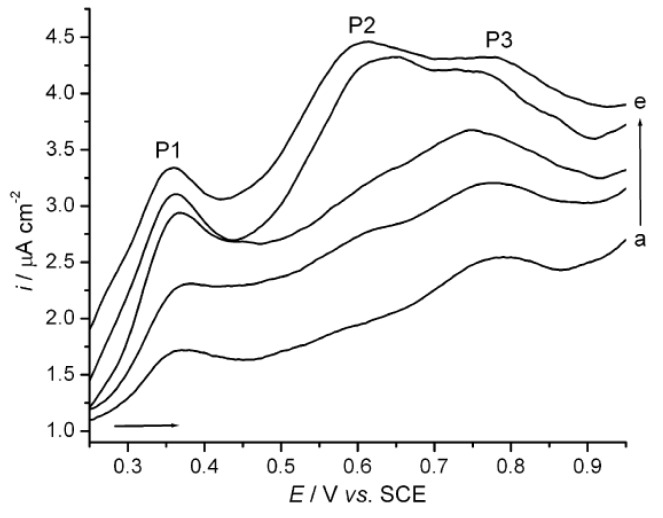
Differential pulse voltammograms of red wines: (**a**) Frankovka, (**b**) Pinot noir, (**c**) Zweigelt, (**d**) Plavac Hvar, (**e**) Ivan Dolac, diluted 1/400 in acetate buffer solution pH3.6, measured at the GC electrode. Scan rate, 5 mV s^−1^, from Seruga *et al*. [49] (with permission from Elsevier).

In recent times, different electrochemical methods have been proposed for the characterization and quantification of polyphenols in wine on the basis that practically all polyphenolic compounds present in wines are electrochemically active. Indeed, the antioxidant properties of these compounds are related to their ability to donate electrons. Thus, most of them present native electroactivity and their electrochemical oxidation at moderate potentials has been exploited for their detections. These characteristics allow selective detection of polyphenols with good sensitivity, even in very complex samples, such as wine, and responses are independent of the optical path length or their turbidity [44]. Cyclic voltametry (CV) was the first electrochemical method used for characterization of polyphenols and determination of total polyphenols content in wine. Indeed, Kilmartin and co-workers first employed this technique in order to characterize a range of phenolic acids and flavonoids, ascorbic acid and sodium metabisulfite, which make an important contribution to the antioxidant properties of wine [45]. These authors indicate that glassy carbon electrode was the best one for this purpose because it minimizes interferences from ethanol which oxidizes at inert metal electrods such as platinum and gold. They showed that the presence of voltammetric signals at low overpotentials was correlated with the presence of polyphenolics of high antioxidant activity, whereas those compounds with low antioxidant power showed electrochemical activity at more positive potentials. Effectively, easily oxidized *ortho*-diphenols yield a low potential peak around 400 mV, the anthocyanins in red wine yield a peak at 650 mV, and harder-to-oxidize functional groups produce higher potential peaks, providing facile discrimination between these types of substrates [45,46]. In another study, De Beer *et al*. attempted to compare the phenolic compounds determination obtained by different methods [47]. They used CV under the same conditions as the Kilmartin group and calculated the total phenol content for wines from integrating the area under the peak to 500 mV (Q_500_) in comparison with the response of catechin standards at 0.01, 0.02 and 0.05 mM. The authors mentioned that using CV to measure total phenols using Q_500_ presented the disadvantages by only reflecting the total content of phenolic compounds containing pyrogallol, gallate, and catechol groups in monomers, oligomers or polymers such as flavanols, proanthocyanidins, flavonols and phenolic acids. Indeed, major anthocyanins but also white wine phenols only produce anodic peaks at potentials higher than 500 mV and are not included in this measurement. However, it is complicated to quantify these phenols at higher potentials since products of polyphenol oxidation accumulate at the electrode surface at higher potentials. A mean to overcome this technical difficulty may be necessary to quantify total current at higher voltage. Nevertheless, they found that CV provided qualitative and semi-quantitative information about polyphenols content and good correlations between the different methods were achieved (Folin Ciocalteu, RP-HPLC and CV). Recently, Sanchez Arribas and co-workers presented an improvement of CV by modification of glassy carbon electrodes with a multi-walled carbon nanotube and coupling with a flow injection analysis [48]. They showed the excellent performance of their modified electrodes under dynamic regime, allowing higher sensitivity and stability as illustrated by the amperometric detection of total polyphenols in wines. Authors mentioned that it is actually possible to use this device for real application in routine analysis where stable and robust responses are required during long period of time [48]. Differential pulse voltametry (DPV) has also been explored in the analytical detection of polyphenols in foods until Seruga *et al*. attempted to apply it to wines [49]. They performed a complete study by DPV, HPLC and spectrophotometric methods in 11 Croatian red wines. By DPV, they noticed three major oxidation anodic peaks on the wine voltammograms (P1; P2; P3) (Figure 3). The first oxidation peak was very well pronounced and it was proven to be the most reversible, reproducible and linear towards catechin standard. The current density of this peak was used for quantification of the total polyphenol content. The second and third oxidation peaks were more or less pronounced and related to the wine origin regions. Finally, Seruga and co-workers showed that DPV was a very sensitive and very selective method for the determination of total polyphenol content of red wines and is a notable improvement for total polyphenol determination in comparison to the cyclic voltametry (CV) and Folin Ciocalteu methods. To put in a nutshell, voltametry techniques have become alternatives to traditional spectrophotometric tests and appear to be very rapid compared with traditional method. However, more experience is required to ensure reproducible and consistent results.

Infrared (IR) spectroscopy is another powerful, fast, accurate and non-destructive technique. This is an alternative to conventional chemical analyses particularly interesting for real-time monitoring of various components during winemaking process as well as for the following of grape maturity. Thus, several studies performed on different grape varieties have shown that Near Infrared (NIR) and Mid Infrared (MIR) spectroscopies combined with multivariate analyses were suitable to evaluate the evolution of the main chemical parameters involved in wine fermentation as well as phenolic compounds (total phenolic, total tannins and total anthocyanins) independently of the constant changes of matrixes during winemaking conditions and giving crucial information about the quality of the final product [38,50,51,52].

Use of NIR spectroscopy was also performed for determining phenolic compounds in skins, seeds or intact berries. For examples, Ferrer-Gallego *et al*. recently determined the concentrations of the main phenolic families (flavanols, anthocyanins, flavonols and phenolic acids) and total phenolic compounds in grape skins and intact red grapes during ripening [53]. They managed to develop models with chemometric tools allowing accurate concentration determination and mentioned that the best results were generally obtained directly recording the spectra of intact grapes attributable to the practical absence of manipulation of the sample that is needed [53]. The same authors have also published an interesting study on the possibility of using NIR to evaluate the monomeric and oligomeric flavanol composition of seeds [54]. In the same perspective, Rolle and co-workers attempted to develop a rapid method for evaluation of total phenol content in intact grape seed by Fourier Transform-Near Infrared spectroscopy (FT-NIR). They obtained promising results but underlined the need to improve their prediction models. Finally, infra red spectroscopy has been used directly in grape berries in order to determine total anthocyanins as well as extractable anthocyanins (pH 1 and 3.2) and total phenols [55]. They reported conflicted data between grapes varieties; anthocyanins extractable at pH 1.0 and pH 3.2 were well predicted by the NIR spectra of intact whole berries in the case of Syrah grapes, whereas they cannot be predicted in other varieties like Cabernet-Sauvignon, Merlot and Carmenère. Furthemore, Cozzolino *et al*. also showed unsuitable low values of R for the estimation of total anthocyanins in intact grape berries [56]. To conclude, the development and application of infrared technique could become an accurate and efficient tool to aid decision making at harvest time and to follow wine process. Nonetheless, harmonization of statistical treatment and chemical methods used for calibration as well as the collection of an important diversity of grapes or wines (various production area, various grape varieties, different maturity stages, different vintages for wines) used for calibration appeared to be primordial to acquire a constant robustness.

### 4.2. Separation and Analysis of Phenolic Compounds (Chromatographic Techniques)

HPLC techniques are widely used for both separation and quantification of phenolic compounds. Various supports, mobile phases, columns and detectors are available for the analysis of anthocyanins, procyanidins, flavonols, flavan-3-ols, and phenolic acids. Several reviews have already focused on these aspects in foods [31,39,57,58,59] and we will pay more attention to recent advances concerning grape and wine analyses.

#### 4.2.1. Sample Preparation

In wines, a prior sample preparation step is sometimes necessary because of the great complexity of chromatograms. Liquid-liquid extraction and solid phase extraction are the most widely used to simplify the chromatograms of wines sample. Porgaly and Büyüktuncel have showed that liquid-liquid extraction of wine with ethyl acetate at pH 2 really improved their data [60]. On the contrary, in many works, wines are filtered and injected without any other preparation. The resolution of the principal compound peaks always appeared really acceptable [18,47,49,61]. Concerning grape or pomace extracts, a simple solubilization of dry powders in appropriate solvents, followed by a filtration is required before injection [28,32].

#### 4.2.2. Columns

Reversed-phase (RP) LC on C18 or equivalent stationary phase is the most common method used for the separation of anthocyanins as well as major phenolic compounds (proanthocyanidins, flavonols, phenolic acids) [16,57,59,62]. These columns are generally packed with spherical particles of silica bonded with octadecyl chain (C18). Unfortunately, chromatographic analyses often take excessive time and sometimes must be preceded by a time-consuming cleanup step. In this perspective, non-conventional monolithic supports (continuous bed) for column packings have been proposed in phenolic analyses [63]. The characteristic feature of non-particulate materials is a continuous character of the skeleton, which fulfils the separation chamber. Due to their rigid and porous structure, they enable higher solvent flows, shorter assay times and fast column re-equilibration between runs. Several studies evidenced the advantages of such kind of columns. For instance, Castellari *et al*. reported that monolithic column could operate at a higher flow-rate than a conventional RP column with a reduced pressure drop and shorter washing and re-equilibration time [64]. A faster separation (36 min) of the monomeric phenolic compounds and an improvement in signal/noise ratio was achieved. Later, Liazid *et al*. developed a more rapid method (8 min) allowing the separation and quantification of 13 common wines polyphenols by using a RP-18e monolithic column [65]. They showed that their method provided reliable, high resolution and reproducible results and could be applied to real samples containing different families of phenolics.

Reversed-phase LC methods can provide specific information on various classes of phenolic in red wines but are limited in their ability to analyze high-molecular mass material. Kennedy and Waterhouse have proposed the use of normal-phase chromatography method (NP-LC) that enables the analysis of high-molecular proanthocyanidins in presence of the anthocyanins [30]. They developed a new method using a silica normal phase column and gradient elution with mobile phase of methylene chloride, methanol, formic acid and heptanesulfonic acid, without extension purification. Based on the elution order of proanthocyanidins and anthocyanins, phenolics elute in order increasing molecular mass. In their comparison of diverse analytical methods of phenolics determination, De Beer and co-workers underlined that NP-HPLC was the most common method for polymer quantification, as polymers for different molecular mass can be separated [47]. On the other hand, for specific favan-3-ols analysis, this technique is less appropriate since the “monomer” peak also includes many nonflavanol monomers. The same limits are shown for anthocyanins monomer determination for the reason that individual compounds are altogether included in one peak. Nevertheless, NP-HPLC can be a useful tool for discriminating age of wines since both monomer and low molecular weight polyphenols contents were noticed to decrease in relation with wine age [47].

The use of a novel mixed-mode ion exchange reversed phase column was also reported by Vergare *et al*. [66]. These particular columns, constituted by a basic group with positive charge embedded in a hydrophobic chain showed promising results for the separation of anthocyanins, including pyranoanthocyanidins in young and aged Cabernet-Sauvignon wines and other varieties [66]. In this study, Vergara *et al*. showed that anthocyanins and pyranoanthocyanins presented different elution order and selectivity in comparison to those obtained in C-18 stationary phase. In spite of a time of analysis nearly twice as long as classical analysis, this technique enables a clear separate elution of first the anthocyanins monoglucosides, then their acetyl-derivates, followed by their coumaroyl-derivates. Pyranoanthocyanins eluted between the coumaroyl-derivates and finally the peak of polymeric compounds, which appears to be correlated with wine age and variety [66].

Finally, core-shell columns have been experimented in order to analyze the phenolic profile of juices and wines produced from interspecific hybrid grape cultivars [67]. As an alternative to UHPLC (ultra high performance liquid chromatography), core-shell column technology is designed to operate on standard HPLC instruments under most operational circumstances. In these works, several methods via core-shell column were assessed to reach the best analyses of atypical phenolic components such as diglucoside-modified anthocyanins found in hybrid cultivars. They showed that C18 and PFP (pentafluorophenyl) core-shell columns resulted in a dramatically improved selectivity, resolution and throughput in each matrix tested. Thus, for anthocyanin/anthocyanidin, monomeric non-anthocyanins compounds or condensed tannins after phloroglucinolysis, the core-shell protocols offer a rapid throughput design, lower solvent consumption, low detection levels and high reproducibility [67].

#### 4.2.3. Detection-Identification of Phenolic Compounds

Various detection methods have been applied in conjunction with HPLC for phenolic compounds determination. Wine phenolics are commonly detected using UV-vis (ultra violet visible), photodiode array (DAD), fluorescence and mass detectors but UV detection remains the most commonly used [59,62] because of the natural absorbance of phenolic compounds in the UV region. Indeed, anthocyanins show two bands absorption maxima in the 265–275 nm and 465–560 nm regions while flavanols show two bands at 210 nm and 278 nm [57]. These properties are used and detection and quantification of these compounds is currently done between 520 nm and 546 nm for anthocyanins and at 280 nm for flavanols [32,59]. However, UV detection is not specific for proanthocyanidin in the presence of other polyphenols.

**Figure 4:**
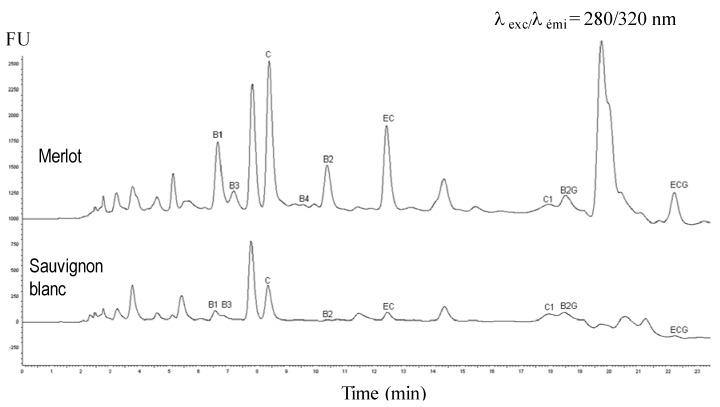
HPLC-Fluo chromatograms of a Merlot and a Sauvignon blanc wine. C: (+)-catechin, EC: (−)-epicatechin, ECG (−)-epicatechin-3-*O*-gallate, B1: procyanidin dimer B1, B2: Procyanidin B2, B3: Procyanidin dimer B3, B4: Procyanidin dimer B4, C1: Procyanidin trimer C1, B2G: procyanidin dimer B2 gallate. Adapted from Silva *et al*. (2012) [61].

Alternative methods include fluorescence detection have been proposed. By combining absorptiometric and fluorimetric detectors, Porgaly and Büyüktuncel managed to simultaneously determine 14 phenolic compounds in red wine and to discriminate overlapping peaks which are frequent in wine analysis [60]. Guerrero also used a combination of UV-detection (320 nm) and fluorescence signals (excitation wavelength = 290 nm, emission wavelength = 320 nm) for their specifity towards hydroxycinnamic acids and flavan-3-ols, respectively [25]. In our team, Silva *et al*. presented a rapid and simple method for the quantification of flavan-3-ols in wine. By fluorimetry, they were able to quantify flavanol monomer, dimer and trimer (excitation wavelength = 280 nm, emission wavelength = 320 nm) as well as (−)-epicatechin gallate and procyanidin B2-gallate, two compounds never quantified by this detection until now with others excitation/emission wavelenghts (270 nm/ 377 nm) (Figure 4) [61].

Structural information for identification of phenolic compounds can be obtained using mass spectrometry (MS). The development and the availability of effective liquid chromatography-mass spectrometry (LC-MS) and the multiple mass spectrometry (MS/MS and MS^n^) systems supplied very useful tools to study the polyphenol structures as well as the mechanisms in which they are involved in winemaking and aging [58]. ESI (electrospray ionization) is a particular effective and the most used technique for the analysis of flavan-3-ols and anthocyanins in the positive or negative-ion modes. This ionization mode has become a good alternative to FAB (fast atom bombardment) used at the beginning of the 1990s, demanding necessarily pre-purification and dissolution of sample in a polar matrix [31]. Thus, in 1997, Cheynier *et al*. studied tannins (oligomers and polymers of flavan-3-ols) in grape seed extracts by LC-MS system equipped with an ESI source operated in the negative-ion mode and a quadrupole mass analyzer [68]. They determined a series of peaks attributed to non-substituted procyanidins from trimers to hexadecamers and their acylated derivates that contained one, two or three gallic acid residues. Recently, NP-HPLC-ESI-MS allowed to Nunez *et al*. to identify non-galloylated and monogalloylated flavan-3-ols up to octamers, and di- and trigalloylated flavan-3-ols up to heptamers in seed extracts from grapes of Graciano, Tempranillo and Cabernet-Sauvignon [69]. They observed 10-fold lower concentrations of monogalloylated flavan-3-ols than non-galloylated flavan-3-ols: this apparent lower response was consistent across samples but it could result from a decreased ionization efficiency of the galloylated forms as compared to the non-galloylated forms. However, because of the lack of galloylated standards, this hypothesis cannot be tested. Based on relative comparisons of the levels found, it was shown that the distribution of flavan-3-ols in seeds was largely determined by genetic factors, and also influence by climatic conditions [69]. Other recent works combining ESI-MS and ESI-tandem (MS/MS) were performed on the identification/quantification of galloylated procyanidins in grape seeds [70]. Indeed, tandem mass spectrometry enables to obtain more specific structural information on a particular compound by a two stages procedure of mass analysis. The ions of interest, issued from the first ionization step are isolated by their characteristic *m/z* values and then re-fragmented and examined in a second step. Tandem MS can be done in two mass spectrometers assembled in tandem (ex: two quadrupoles) or in a single mass analyzer capable of storing ion (ex: quadrupole ion trap) [71]. In Guerrero *et al*.’s works, MS-MS fragmentation was effective for differentiating some compounds such as quercetin-3-glucuronide and isorhamnetin-3-glucoside which present the same mass and the same first parent ion (*m/z* = 477) but were distinguished by the ion after MS^2^ (301 for quercetin-3-glucuronide and 315 for isorhamnetin-3-glucoside) [25]. Really recent (2012) work from Delcambre and Saucier evidenced the performance of UHPLC in combination with ESI and Q-TOF (quadrupole time of flight) in targeted mode MS/MS for determining new structures [18]. Time-of-flight mass spectrometry is based on a simple mass separation principle. Considering ionized species starting from the same position at the same time and being accelerated by means of a constant homogeneous electrostatic field, their velocities are unambiguously related to their mass-to-charge ratio and times of arrival at a detector directly indicate their masses. The time-of-flight instrument possesses a number of extraordinary advantages over most other types of mass analyser. Theoretically, this technique presents unlimited mass range since it needs no ion beam scan, allowing the obtaining of a complete mass spectrum for each ionisation event. High transmission, sensitivity and the necessity of extremely small sample amounts (<10–18 mol in the most modern instruments) are the others known advantages of this technique [72]. Thus, Delcambre and Saucier partially identified for the first time 14 monomeric flavanol glycosylated compounds based on four aglycons ((+)-catechin-, (−)-epicatechin-,(−)-epigallocatechin- and epicatechin gallate monoglycosides) in red wine and grape seed extracts by targeted ESI-MS/MS [18]. The targeted MS/MS mode provided additional information about the structures of these compounds. The fragments resulting from the fragmentation of these compounds provide a specific signature that allows a better identification. Nevertheless, this method, based on exact mass and specific fragmentation pattern could not provide information on the exact position of glucose.

The coupling of HPLC with mass spectrometry was also a key development to gain structural information and structure elucidation of anthocyanins, anthocyanidins and new coloured class of anthocyanin derivates. The five common anthocyanins in the grape from *Vitis vinifera* are delphinidin (Dp), cyanidin (Cy), petunidin (Pt), peonidin (Pn) and malvidin (Mv), present at 3-*O*-monoglucosides, 3-*O*-acetylmonoglucosides and 3-*O*-(6-*O*-*p*-coumaroyl)monoglucosides. In 2010, He *et al*. confirmed the existence of a sixth anthocyanin skeleton, pelargonidin-3-*O*-glucoside in non-teinturier *V. vinifera* grapes (Cabernet Sauvignon and Pinot Noir) [73]. In the not *Vitis vinifera*, anthocyanins with a second glucose linked to the C-5 hydroxyl group may be present [74]. Recently, De Freitas and Mateus have reported the diversity of pyranoanthocyanins in wines. The formation of these anthocyanin-derived pigments naturally occurs in red wine aging and a variety of structures (namely carboxypyranoanthocyanins, methylpyranoanthocyanins, pyranoanthocyanin-flavanols, pyranoanthocyanin-phenols, portisins, oxovitisins and pyranoanthocyanidin dimmers) was identified in the two last decades [75]. Operating generally in the positive ion mode and in acidic medium, anthocyanins are detected as flavylium cations, M^+^ to provide signals of high intensity [58]. By coupling HPLC and an electrospray interface with a mass detector, sixty-six different anthocyanins were detected in a *Vitis vinifera* Dornfelder red wine from 1997 vintage [76]. These anthocyanins were assigned to the well-known mono- and diglucosides (acetyl- and coumaryl-forms) of wines. In addition, the acetic acid esters and coumaric acid esters of the 3-glucosides were detected. Aging products such as vitisin A (*m/z* = 561) and vitisin B (*m/z* = 517) and acetylvitisinA (*m/z* = 603) and B (*m/z* = 559), already identified in other works were also identified in this wine [77]. Fulcrand *et al*. showed that vitisin A resulted from cyclization between carbon 4 and the 5-hydroxyl group of the original anthocyanin flavylium moiety with the double bond of the enolic form of pyruvic acid, followed by dehydratation and rearomatization steps [78]. Finally, by applying an acidic hydrolysis to the wine, Heier *et al*. indicated that all pigments detectable at 525 nm were derived from the skeleton of delphinidin, cyanidin, peonidin, petunidin, malvidin and their derivates of pyruvic acid and acetaldehyde [76]. More recently, Xu and co-workers attempted to develop a rapid and accurate method for anthocyanidin quantification in grapes and grape juices through an assisted hydrolysis using LC/MS [79]. Under optimized conditions, the five major anthocyanidins (Dp, Cy, Pt, Pn, Mv) were fully separated within 25 min and successfully quantified. This method was proposed as a global approach to quantify anthocyanidins in order to avoid the difficulties of separation/identification of the wide variety of anthocyanins present in grape matrices and wines. After fractionation of the pigment material of red wine, the existence of dimeric anthocyanins (A-A^+^), previously detected in grape skin was shown by Alcalde-Eon *et al*. [80]. Thanks to the employment of mass detection in series (MS^n^; n = 1–4), new oligomers were detected in wine. They consisted of a flavanol (catechin, epicatechin, gallocatechin or epigallocatechin), linked through its C_4_ position to the nucleophilic positions of the upper units of a dimeric anthocyanin (F-A-A^+^). All the identified compounds contain malvidin as one of the anthocyanin subunits, whereas the other anthocyanin moiety could be either Dp, Cy, Pt, Pn or Mv. This study was an interesting example of the utility and power of ESI/MS^n^ analysis coupled or not to HPLC [80].

In de Villiers *et al*.’s study, optimal chromatographic conditions were developed and applied to a 2-year old Pinotage wine, to allow the separation and identification of more than one hundred anthocyanins and derived pigments in a single run [81]. Thus, by this procedure, these authors tentatively identified 17 common anthoycanin-glucosides and -diglucosides, seven oligomeric anthocyanins, 24 direct anthocyanin-tannin adducts such as Mv-glc-(epi)gallocatechin (A-Ttype), nine acetaldehyde-mediated tannin aducts, 10 anthocyanin-vinylflavanol condensation products, eight anthocyanin-vinylphenol condensation products (pyranoanthocyanins), 12 anthocyanin-pyruvic acid products (vitisin A derivatives), five anthocyanin-acetaldehyde derivatives (vitisin B derivatives) and 2 anthocyanin-acetone derivatives.

**Figure 5:**
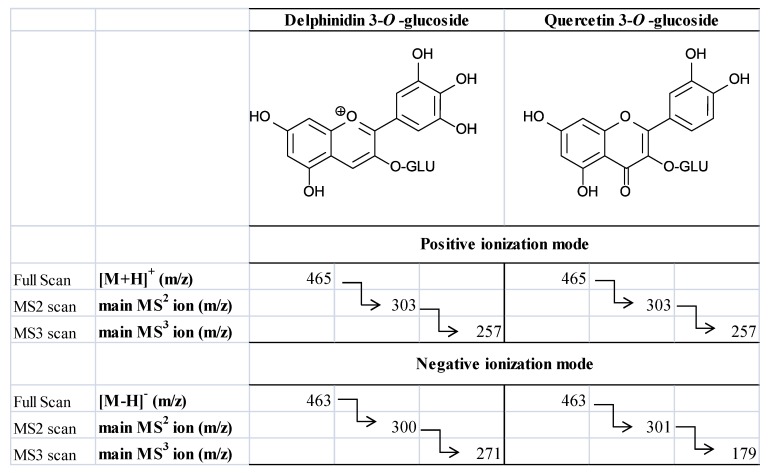
Comparison of the full scan and MS^2–3^ scan of delphinidin-3-*O*-glucoside and quercetin-3-*O*-glucoside in positive and negative ionization mode. Adapted from Sun *et al*. 2012 [83].

Acevedo de la Cruz and co-workers have recently shown the efficiency of combination of mass spectrometry and NMR spectroscopy resulting in the identification of 33 anthocyanins in four different Vitis species [82]. In particular, newly reported *cis* isomers of *p*-coumaric-derivatives were identified (petunidin-, peonidin- and malvidin-3-(6-*p*-coumaroyl)-5-diglucoside). Finally, really recent works porposed an alternative to classical positive ionization mode by applying negative mode for characterization of anthocyanins [83]. In positive mode, flavonol glycosides, e.g. quercetin glycosides, possess the same molecular ions and fragmentation patterns as the corresponding anthocyanins, e.g., delphinidin glycosides ([M]^+^ of anthocyanins and [M+H]^+^ of flavonol glycosides) are the same. Reversely, the MS spectra acquired in the negative ionization mode proved to be a valuable tool for differentiation of anthocyanins from non anthocyanin polyphenols (Figure 5). Specifically, the doublet ions of [M−2H]^−^ and [M−2H+H_2_O]^−^ were unique to anthocyanins while a single molecular ion [M−H]^−^ dominated the spectra of non-anthocyanin polyphenols [83].

## 5. New Separation Techniques

Still motivate by gaining time, new systems such as UPLC (ultra-high performance liquid chromatography) appeared recently to overcome some of the LC drawbacks and offering some clear benefits in terms of analysis time, resolving power, solvent consumption, and to a better extent sensitivity [18,81,83]. Indeed, after a polyphenol extraction from wines by SPE on a new hydrophilic-lipophilic balanced sorbent, Silva *et al*. managed to separate and analyzed, in five minutes, fifteen phenolic compounds mainly belonging to flavonols, flavanols and phenolic acids [84].

Capillary electrophoresis (CE) has received significant attention as an alternative liquid-based separation method to HPLC since the 1990s. It is especially suitable for the separation and quantification of low to medium molecular weight polar and charged compounds, the resultant separations being often faster and more efficient than the corresponding HPLC separations. CE permits the simultaneous analysis of analytes with different nature in a single run. In wines, the most representative revised compounds are phenolic compounds, amino-acids, proteins, elemental species, mycotoxins and organic acids [85]. However CE methods generally suffer from lower sensitivity and robustness compared to standard HPLC methods, and partially for these reasons, the technique has primarily found application in certain niche-areas where CE provides clear benefits compared to HPLC (for example chiral separation) [62]. Concerning analysis of phenolic compounds in wines, the comparison of capillary zone electrophoresis-UV-vis-MS with RP-LC-UV-ESI-MS for the analysis of monomeric phenolic compounds in extracts of red wines showed that RP-LC remains the method of choice for phenolic analysis [85,86]. Very few reports have appeared on the application of CE methods for the separation of anthocyanins and no direct application onto wines was reported probably because of the absence of improvement of the separation of complex samples in comparison with LC techniques [59].

Because of the importance of developing clean chemistry procedures, emerging methods for food matrices are based on solvent-free procedures. In this perspective, Vinas and co-workers paid attention to the development of a solid-phase microextraction (SPME) GC-MS method for the analysis of some polyphenols in wine and grapes [87]. Direct immersion SPME was used for the adsorption of polyphenols and then the fiber was placed in the headspace of the derivatizing reagent, BSTFA bis(trimethylsilyl)trifluoroacetamide, necessary to convert the polar non-volatile compounds into volatile derivates. These authors developed a new sensitive method for the determination of both cis- and trans- resveratrol isomers, piceatannol, catechin and epicatechin. Nevertheless, this technique can be only useful for such low molecular weight compounds even if interpretation of fragments patterns appears more difficult because of the derivatization. Indeed, the derivatization, required for this technique, increase the molecular weight of phenolic compounds and this could results in high molecular weight exceeding the mass rank available for the most common GC/MS systems, thus making this approach ineffective.

## 6. Techniques to Establish Tannin Structures (Mean Degree of Polymerization)

**Figure 6:**
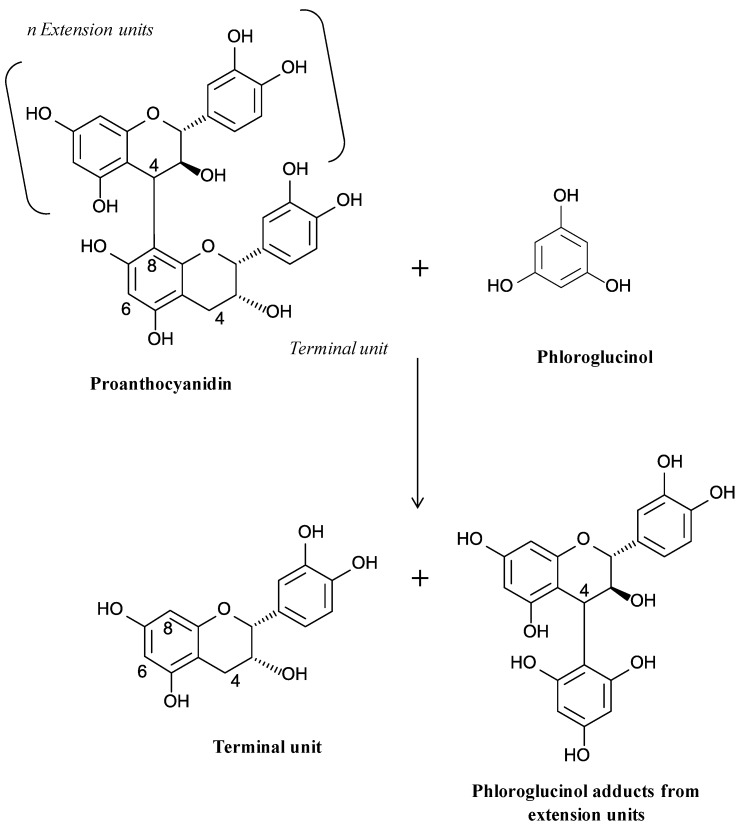
Reaction pathway of phloroglucinolysis.

Interest in the characterization of condensed tannins has increased in the last decades. Proanthocyanidic tannins may differ by the nature and the number of constitutive units, by the type and location of interflavanic linkages connecting the monomeric unit (C4-C8 or C4-C6) and lastly by their conformation (linear versus branched) of polymers. The characterization of condensed tannins by depolymerisation is often employed [31]. Treatment of a condensed tannin with acid, in the presence of a nucleophile such a thiol or less putrid and therefore much preferred phloroglucinol allows the subunit profile to be analysed by HPLC and the average molecular mass (expressed as “mean degree of polymerization, mDP”) to be calculated [88,89] (Figure 6). Indeed, proanthocyanidins become depolymerized, releasing terminal subunits as flavan-3-ol monomers and extension subunits as electrophilic flavan-3-ol intermediates. The electrophilic intermediates can be trapped by the nucleophilic reagent to generate analyzable adducts. Most of our current knowledge about general composition and structure of grape and wine tannins has been obtained by depolymerization. Grape seeds proanthocyanidins comprise only procyanidins (sub-units constituted of (+)-catechin and (−)-epicatechin) whereas grape skins proanthocyanidins include both procyanidins and prodelphinidins (sub-units constituted of (−)-epigallocatechin) [15,90].

Skin proanthocyanidins have a higher mDP and a lower proportion of galloylated sub-units than seeds ones in major of the varieties [16,28,91,92]. Some authors have applied these depolymerization methods to the wines [93,94,95]. Especially, Drinkine *et al*. adapted the phloroglucinolysis to ethylidene-bridged flavan-3-ols analysis which result from chemical modifications of flavan-3-ols occurring during wine making and aging. By this method, the authors showed that flavan-3-ol ethylidene bridges represented less than 4% of flavan-3-ol bonds and that the proportion of these linkages relative to native interflavan bonds increased with wine age. Nevertheless, Herderich and Smith underlined that this technique is limited for the characterization of wine tannins since the vast majority of wine tannins resists the acid mediated depolymerization, allowing only a minor portion of tannin to be characterized [31]. These depolymerization methods are difficult to implement and do not give information about the polymer distribution of a tannin fraction because all the polymers contained in the fraction are cleaved into monomer units in the course of the reaction. For example, when studied the depolymerization of a sample containing a mix of 50% of small tetrameric tannins and 50% larger octametric tannins, will yield an mDP of six, the same as considering a sample consisting of 100% hexameric tannins.

Several studies have focused on the separation of tannins according to their size for analytical and preparative purposes. Concerning the analytical aspect, convenient separation can be achieved by NP HPLC as previously described [30]. For preparative aspects, gel chromatography with different gels such as TSK HW-40 have been used [33,96]. Unfortunately, only oligomers up to five are easily separated with these methods. Saucier *et al*. proposed a rapid fractionation of grape seed proanthocyanidins based on liquid/liquid extraction and relative solubility of these compounds in different solvents (water, ethyl acetate, methanol, and chloroform) [29]. By this method, they were able to obtain 6 fractions having mDP varying between three and 13, approximately, and they can be easily obtained in gram quantities which may be useful to study the properties of each fraction. Recently, Hanlin *et al*. employed semi-preparative liquid chromatography on a diol phase column to fractionate grape seed, skin and wine proanthocyanidins [97]. They obtained an effective fractionation conducted in interesting trends of proanthocyanidins distribution in Cabernet-Sauvignon and Shiraz varieties. The extensive fractionation leads to the obtention of some skin fraction up to mDP of 76 for Cabernet-Sauvignon skins. Recently, mass spectrometry (MS) was proved to be an interesting alternative technique allowing condensed tannins to be detected without sample pre-treatment. Mouls *et al*. attempted to better define the difficulties encountered in the MS analysis of tannins [98]. For this purpose, they compared mDP values obtained after chemical depolymerization and mass spectra data processing of tannin extracts from apple cultivars. They assessed the impact of several analytical parameters (solvent acidity, ionization mode, cone voltage) and showed that they strongly affected the mass spectrometric responses of tannins. Nevertheless, the mDP values calculated from mass spectra were still underestimated in comparison to mDP for depolymerization, underlining the difficulties in detecting high mDP tannin. They concluded that ESI-MS was suitable for mDP estimation of fractions with low molecular weight, displaying a narrow polymer distribution with standard mDP below 20.

## 7. Conclusions

Hundreds of publications on the analysis of grape and wine phenolic compounds have already appeared over the past four decades. New analytical techniques have unraveled some structures derived from tannins and anthocyanins in wine and determined how they are formed. Thus, the diversity of methods and experimental procedures reflects the complexity of phenolic analytes in grape and in wine. Improvement is still pursued since each species is present in very small amounts and too many unidentified compounds still remain, especially with the polymeric fraction. Considering environmental problems, several novel extraction and analyses techniques have been developed as an alternative to conventional extraction and analyses procedures, offering advantages with respect to analysis time, solvent consumption, extraction yields and reproducibility. Nonetheless, there is still no available standardize procedure for sample preparation, extraction and analyses. Each method provides specific advantages, detections towards some particular phenolic compounds and these aspects have to be thought before analysis. Choosing an appropriate phenolic assay depends on what information is required. Depending on the needs of an experiment, using some combination of assays is currently the best approach to properly characterize the phenolic composition of a sample.
